# Supplementary material for: Description of Antimicrobial-Resistant Escherichia coli and Their Dissemination Mechanisms on Dairy Farms
Source: Vet Sci. 2023 Mar 23;10(4):242. doi: 10.3390/vetsci10040242 (PMC10144642; doi:10.3390/vetsci10040242)
Supplement: Supplementary file 1 [file vetsci-10-00242-s001.zip › Supplementary Table S3.pdf]

Supplementary Table S3. Correlation between phenotypic and genotypic resistance for various Minimum Concentration Inhibition (MIC) values

Antimicrobials	MIC Values		
	Breakpoints[1]	Epidemiological cut-off values[2]	Best fitting MIC[3]
Amoxicillin-clavulanic acid	8	NONE	16
Ampicillin	8	8	4–32
Cefoxitin	8	8	16
Ceftriaxone	1	0.25	2–4
Ceftiofur	2	1	2–4
Meropenem	1	0.064	0.064
Gentamicin	4	2	2–4
Neomycin	8	8	8
Streptomycin	32	16	8
Spectinomycin	32	64	16
Chloramphenicol	8	16	16
Florfenicol	NONE	16	8
Nalidixic acid	16	8	16
Ciprofloxacin	0.25	0.064	0.032
Danofloxacin	0.25	NONE	0.125
Enrofloxacin	0.25	0.125	0.125
Azithromycin	16	16	8
Sulfisozaxole	256	NONE	64–128
Trimethoprim- sulfamethoxazole	2	0.25	0.25
Tetracycline	4	8	4–8
Sensitivity	97	95	99
Specificity	93	94	97
Overall agreement	95	94	98

All MIC values in the table represent the highest concentration for a susceptible phenotype; [1]Breakpoints (intermediate considered as resistant) according to CLSI M100 [22] (*Enterobacterales* : amoxicillin/clavulanate, ampicillin, azithromycin, cefoxitin, ceftriaxone, chloramphenicol, ciprofloxacin, gentamicin, meropenem, nalidixic acid, sulfisoxazole, tetracycline and trimethoprim/sulfamethoxazole), CLSI VET01S [23] (Bovine Respiratory pathogens : ceftiofur, danofloxacin, enrofloxacin and spectinomycin), or CIPARS [24] (streptomycin). A breakpoint was not available for neomycin, thus the epidemiological cut-off value from EUCAST was used (MIC $\geq$ 16 µg/mL was defined as resistant). [2] Values from EUCAST to differentiate wild-type and non wild-type isolates; [3]Minimun inhibition concentration that best match the resistance genes found for isolates describe in this study (n=118)
